# Role of psychiatric hospitals during a pandemic: introducing the Munich Psychiatric COVID-19 Pandemic Contingency Plan

**DOI:** 10.1192/bjo.2020.167

**Published:** 2021-02-01

**Authors:** Kristina Adorjan, Oliver Pogarell, Dorothee Streb, Frank Padberg, Christian Erdmann, Gabriele Koller, Florian Raabe, Daniela Reich-Erkelenz, Sylvia de Jonge, Karin Neumeier, Peter Zill, Karl-Walter Jauch, Thomas G. Schulze, Peter Falkai

**Affiliations:** Department of Psychiatry and Psychotherapy, University Hospital, LMU Munich, Germany; Institute of Psychiatric Phenomics and Genomics (IPPG), University Hospital, LMU Munich, Germany; and Center for International Health (CIHLMU), University Hospital, LMU Munich, Germany; Department of Psychiatry and Psychotherapy, University Hospital, LMU Munich, Germany; Department of Psychiatry and Psychotherapy, University Hospital, LMU Munich, Germany; Department of Psychiatry and Psychotherapy, University Hospital, LMU Munich, Germany; Department of Psychiatry and Psychotherapy, University Hospital, LMU Munich, Germany; Department of Psychiatry and Psychotherapy, University Hospital, LMU Munich, Germany; Department of Psychiatry and Psychotherapy, University Hospital, LMU Munich, Germany; Institute of Psychiatric Phenomics and Genomics (IPPG), University Hospital, LMU Munich, Germany; Department of Molecular Neurobiology at Department of Psychiatry and Psychotherapy, University Hospital, LMU Munich, Germany; Institute of Psychiatric Phenomics and Genomics (IPPG), University Hospital, LMU Munich, Munich, Germany; and Department of Molecular Neurobiology at Department of Psychiatry and Psychotherapy, University Hospital, LMU Munich, Germany; Department of Molecular Neurobiology at Department of Psychiatry and Psychotherapy, University Hospital, LMU Munich, Germany; University Hospital, LMU Munich, Germany; Institute of Psychiatric Phenomics and Genomics (IPPG), University Hospital, LMU Munich, Munich, Germany; Department of Psychiatry and Psychotherapy, University Hospital, LMU Munich, Germany

**Keywords:** Mental disorders, SARS-CoV-2 infection, COVID-19, psychiatric hospitals, pandemic plan

## Abstract

**Background:**

Psychiatry is facing major challenges during the current coronavirus disease 2019 (COVID)-19 pandemic. These challenges involve its actual and perceived role within the medical system, in particular how psychiatric hospitals can maintain their core mission of attending to people with mental illness while at the same time providing relief to overstretched general medicine services. Although psychiatric disorders comprise the leading cause of the global burden of disease, mental healthcare has been deemphasised in the wake of the onslaught of the pandemic: to make room for emergency care, psychiatric wards have been downsized, clinics closed, psychiatric support systems discontinued and so on. To deal with this pressing issue, we developed a pandemic contingency plan with the aim to contain, decelerate and, preferably, avoid transmission of COVID-19 and to enable and maintain medical healthcare for patients with mental disorders.

**Aims:**

To describe our plan as an example of how a psychiatric hospital can share in providing acute care in a healthcare system facing an acute and highly infectious pandemic like COVID-19 and at the same time provide support for people with mental illness, with or without a COVID-19 infection.

**Method:**

This was a descriptive study.

**Results:**

The plan was based on the German national pandemic strategy and several legal recommendations and was implemented step by step on the basis of the local COVID-19 situation. In addition, mid- and long-term plans were developed for coping with the aftermath of the pandemic.

**Conclusions:**

The plan enabled the University Hospital to maintain medical healthcare for patients with mental disorders. It has offered the necessary flexibility to adapt its implementation to the first and second waves of the COVID-19 pandemic in Germany. The plan is designed to serve as an easily adaptable blueprint for psychiatric hospitals around the world.

## Background

The disease now referred to as coronavirus disease 2019 (COVID-19), which is caused by the so-called severe acute respiratory syndrome coronavirus 2 (SARS-CoV-2), has posed considerable challenges to the maintenance of medical care for patients with mental disorders. Reduced therapy programmes, infected staff, quarantine periods and isolation – these measures may hinder psychiatric hospitals in their provision of care for patients. In addition, managing in-patients with COVID-19 who have comorbid psychiatric disorders poses several challenges and therefore requires intensive cooperation with internal medicine.

In this setting, some institutions abruptly reduced therapy programmes, cut back on hospital beds or even closed services entirely. These approaches can have fatal consequences for patients with mental disorders. Psychiatric patients are considered extremely vulnerable in the COVID-19 pandemic. People with mental disorders frequently face chronic illness courses and a reduced median life expectancy. They often deal with poverty. Furthermore, their housing options, access to educational institutions and activities and social contacts are often limited because of their poor communication and interpersonal skills. Another challenge that needs urgent attention is the unknown impact of the COVID-19 pandemic on people with mental disorders in terms of symptom severity, relapses and need for increased frequency and intensity of mental healthcare.^1^

Additional stressors, such as curfews, quarantine and isolation, together with the stress of the overall situation during a pandemic, can lead to increases psychological stress, not only in people with mental disorders but also in the entire general population.^1^ For this reason, the operability of psychiatric hospitals must be fully maintained.

## Contingency plan

Maintaining mental health services is the goal of the Munich Psychiatric COVID-19 Pandemic Contingency Plan (MPCPCP), which offers specific recommendations on how to adjust to the pandemic situation. The MPCPCP was developed and put into action for the Ludwig-Maximilians-University (LMU) Munich University Hospital and incorporates recommendations of the National Pandemic Plan, the guidelines of the Robert Koch Institute^2^ and the Supplement to the National Pandemic Plan-COVID-19 in Germany. However, it is designed to serve as an easily adaptable blueprint for psychiatric hospitals around the world.

The LMU Hospital is the second largest general hospital in Germany. The Department of Psychiatry and Psychotherapy is part of the general hospital and is the second largest psychiatric department in Germany. The mutual use of services (for example, transfer of patients, consultation) is possible within the hospital.

The overall goal of the MPCPCP is to establish psychiatric care within the framework of a large maximum care centre (such as the LMU University Hospital) as a fully integrated partner with other disciplines as far as reasonably possible. In addition, the aim of this pandemic contingency plan is to contain, decelerate and, preferably, avoid transmission of COVID-19 (for a synopsis of symptoms, see Appendix 1) in a psychiatric hospital and to enable and maintain medical healthcare for patients with mental disorders. Furthermore, the pandemic plan could provide an example of how a psychiatric hospital can participate in acute care within a healthcare system. The pandemic plan applies primarily to university hospitals or hospitals that facilitate cooperation between psychiatry and internal medicine or tertiary care units.

## Method

The phased MPCPCP was written as a joint effort by K.A., O.P., T.G.S. and P.F., all of whom work at the Department of Psychiatry and Psychotherapy (K.A., O.P., P.F.) and at Institut für Psychiatrische Phänomik und Genomik (K.A., T.G.S.) of the University Hospital of LMU Munich. The plan was completed at the beginning of March 2020 and was adapted step by step as the pandemic situation changed and evolved. When devising the plan, staff considered the national pandemic strategy and several legal regulations, in particular the valuable recommendations of the Robert Koch Institute.

To ensure flexibility and applicability, the developers prepared the plan as phases that build on each other; for example, the measures to be applied in phase 1 are also valid in subsequent phases. Furthermore, they designed the plan in such a way that, depending on the situation, stages can be skipped (for example from phase 3 to phase 5) or returned to. The multidirectional design of the plan was deemed important because the pandemic situation was expected to progress in waves. Right from the start, the developers aimed to further refine the plan on the basis of the latest scientific and clinical experience.

## Results

### The MPCPCP

**Fig. 1:**
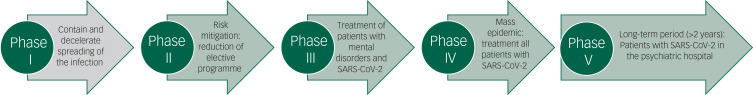
An overview of the Munich Psychiatric COVID-19 Pandemic Contingency Plan – overview. COVID, coronavirus disease; SARS-CoV-2, severe acute respiratory syndrome coronavirus 2.

The MPCPCP consists of five phases that are dictated by the course of the pandemic and a healthcare system's overall readiness to adapt to this course (Fig. 1). The time frame allocated to each phase can differ from region to region and from institution to institution. Also, phases show a high degree of temporal overlap. The MPCPCP's approach and procedures are summaries in Appendix 2.

#### Phase I

In phase I, the focus lies on increasing hygienic measures and raising awareness and readiness among staff members and patients. These activities may include swiftly conducting surveys on SARS-CoV-2 (e.g. screening questions regarding potential contact with people with COVID-19) among the staff and patient population, in particular in out-patient clinics. Limitation of regular therapy sessions is not necessary at this stage. In this period, the focus is placed on the care of patients with mental disorders without SARS-CoV-2 infection. Both in-patient, out-patient and day clinic services can be maintained at their usual frequency and service quality (no reduction of therapy program).

#### Phase II

Once the infection rate in the population increases and general elective medical care is restricted, the hospital enters phase II. Here, the focus lies on a carefully considered reduction of the elective programme to free up resources to treat patients with COVID-19. While measures for further risk mitigation are taken, the duration and content of therapy sessions are adjusted to the current staffing situation.

Hygiene measures in therapy groups (small groups with a maximum of 5 to 6 people, distance between people >2 m) are put in place and psychoeducation about the SARS-CoV-2 infectious disease is integrated into the therapy plan. Moreover, the impact of isolation and social distancing on mental health, the handling of stress situations (such as isolation and loneliness) and the activation of (new) psychological resources are integrated into these psychoeducational measures. At this point particular attention has to be paid to protecting high-risk patients, but the focus still lies on the care of patients with mental disorders without SARS-CoV-2 infection. Maintaining clinical operations and adjusting to the epidemiological situation are the primary aims; patient contacts with therapists should be limited and only take place if urgently indicated and according to recommended hygienic measures.

In out-patient settings, treatment is provided only in emergencies and for patients for whom the possibility of supervision by a psychiatric specialist outside of the hospital is not available. The primary goal of out-patient care in this phase consists of preventing the admission of patients to in-patient care who could well receive treatment in an out-patient unit. Day clinic care is still available.

#### Phase III

As soon as patients with a mental illness and a SARS-CoV-2 infection arrive for treatment, a psychiatric hospital moves into phase III. Phase III of the MPCPCP describes the necessary measures for the treatment of such patients. The Munich University Hospital for Psychiatry and Psychotherapy has converted one of its regular wards into a so-called Psychiatric COVID ward. This ward officially serves the entire LMU University Hospital. Consequently, the psychiatric department is now actively involved in the care and treatment of patients with a disease related to SARS-CoV-2. The Psychiatric COVID ward is run as a separate organisational unit that treats patients with mental disorders and a positive SARS-CoV-2 diagnosis or COVID-19.

##### Preparation of the Psychiatric COVID ward

The Psychiatric COVID ward is equipped with oxygen masks and oxygen access, larynx masks, pulse oximeters, clinical thermometers, FFP2 (respiratory) masks and protective wear. Employees are extensively trained in hygienic measures. Two permanent teams are formed with selected senior doctors, residents and nurses for 14 days each. These teams exclusively treat patients with COVID-19. In the first two weeks, Team A is involved in direct patient care, whereas team B works from home. Administrative medical duties can be executed remotely. In case of employee absence (for example because of illness) in team A, employees from team B can be immediately assigned to patient care. After 14 days, the teams change. This is followed by a 14-day rotation of the teams.

The Psychiatric COVID ward is divided into two areas: the front area is used for patients with suspected cases and the back area for patients with confirmed COVID-19. Swabs for SARS-CoV-2 polymerase chain reaction (PCR) tests are taken daily in a separate room. All affected patients and hospital staff members are tested regularly. Thus, only one person has to put on protective gear for testing, which saves resources. For organisational reasons, samples from patients are tested in an external, certified laboratory. In Munich, samples taken from staff members are tested in the hospital's own laboratory, which has developed its own test methods for employees to relieve the burden on other laboratories and to perform tests faster (see Supplementary data 1 and Supplementary Fig. 1; available at http://dx.doi.org/10.1192/bjo.2020.167).

##### Patients considered for treatment on the Psychiatric COVID ward

**Fig. 2:**
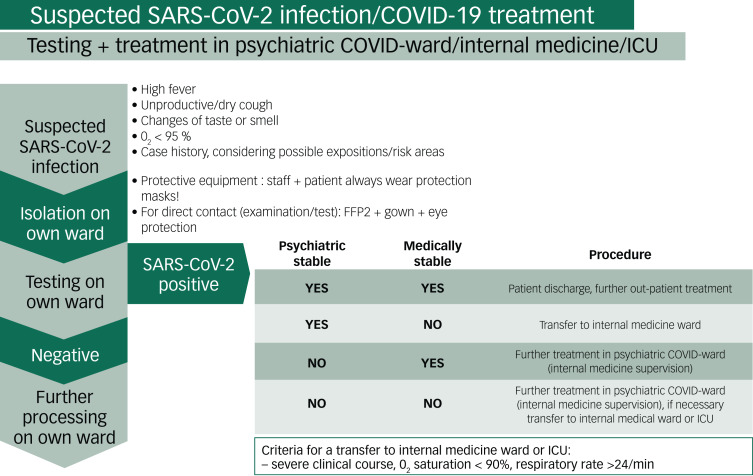
Testing and treatment procedures on the Psychiatric COVID ward. COVID, coronavirus disease; ICU, intensive care unit; SARS-CoV-2, severe acute respiratory syndrome coronavirus 2.

Psychiatric patients who are positive for SARS-CoV-2 and considered for in-patient treatment at a psychiatric hospital are mainly in a stable medical condition but mentally too unstable for out-patient treatment (Fig. 2). However, patients with severe cases should be treated by specialists in appropriate internal or intensive care units. In addition, a re-transfer of patients to a psychiatric unit is also possible, for example in the case of an organic psychiatric disorder because of severe infectious disease, adjustment disorder or other causes.

During phase III, the team of the Laboratory of Molecular Neurobiology at the Department of Psychiatry and of the Institute of Psychiatric Phenomics and Genomics developed their own PCR method for detecting SARS-CoV-2 virus. The development of this test method was supervised by the Institute of Virology of the Max von Pettenkofer Institute. Having in-house testing capability offers the possibility of quickly and adequately testing medical staff members and relieves overstrained microbiological institutions. Within 4 weeks, 114 of a total of 300 employees were tested; 3 were positive.

Asymptomatic patients who have been in contact with individuals with a SARS-CoV-2 infection do not need to be tested for SARS-CoV-2 but must be isolated and wear protective masks accordingly. If the contact patients remain asymptomatic until the 14th day after the last contact with someone who tested positive for SARS-CoV-2, the isolation can end. Asymptomatic staff who have had contact with someone who tested positive but who are still working clinically must be tested for SARS-CoV-2 on day 6. However, no testing is required for asymptomatic staff members who have been in home quarantine for 14 days.

##### Out-patient settings

Out-patient mental health services are also indispensable and systemically relevant in phase III, especially during a pandemic. They may be required after early discharge from in-patient facilities, for example. Emergency services are broadly defined: all patients who require treatment for a mental illness may come to the out-patient clinic at any time. Out-patient opioid maintenance treatment should also be offered. However, special out-patient clinics (for example for attention-deficit hyperactivity disorder, Tourette syndrome) will see some reduction of services.

#### Phase IV

In case of a mass epidemic (increased incidence of SARS-CoV-2 within a very short time) (phase IV) an interdisciplinary admission of COVID-19 patients at the psychiatric department is envisaged. Patient admission is independent of psychiatric diagnoses; in phase IV, the hospital is open to all patients and psychiatry is fully integrated into somatic medicine.

If the Psychiatric COVID ward is full and the number of psychiatric patients with COVID-19 increases further, cohorting is made possible ward by ward. The closed wards are still required to be ‘mixed’ wards for acutely ill patients with and without COVID-19. The care of patients with SARS-CoV-2 infection is now independent of any primary mental illness; seriously ill patients are expected to be supervised. Securing the survival and medical care of patients is paramount. The multimodal psychiatric treatment concept is discontinued and only medical and psychopharmacological care is maintained for patients with mental disorders.

#### Phase V

Phase V is considered as the long-term period (>2 years: duration of the pandemic or until a vaccine is available) in which patients with a mental illness and a SARS-CoV-2 infection are expected to be seen continuously in the psychiatric hospital. As long as there is a risk of a SARS-CoV-2 infection in the population, the Psychiatric COVID ward needs to be maintained. In addition, implementing a psychosocial rehabilitation ward in psychiatric hospitals could ensure adequate aftercare for patients who had severe COVID-19 at an earlier stage or who have developed a mental illness (for example maladaptation, depression) as a result of a serious infectious disease. This step can relieve intensive care units by increasing bed capacity. To prevent the transmission of SASR-CoV-2 infection, some wards can be reserved for the treatment of patients with mental illness only, while other wards can gradually be opened for the care of patients with COVID-19.

### Enabling clinical management of patients with SARS-CoV-2 in psychiatry: the Psychiatric COVID ward

#### Clinical diagnostics

##### Patient admission (suspected or confirmed SARS-CoV-2 infection)

Isolation and protection measures (‘safety first’) were put in place. Upon suspicion of a SARS-CoV-2 infection, protection measures are immediately and stringently required and patients should be isolated. A group isolation (cohorting) of patients is not permitted in principle but can be considered in case of a confirmed SARS-CoV-2 infection. Medical staff members are instructed to reduce patient contact to an essential minimum and patient contact is reserved for trained staff members only. Stringent implementation of basic hygiene measures, including hand hygiene measures, is needed.

Availability of personal protective equipment must be ensured: protective gown (non-reusable, waterproof), disposable gloves, respiratory mask (FFP2) and safety glasses or face shield. Staff members must be trained, for example via video training. The first patient contact should include a short patient interview and a clinical examination as follows:

(a) case history, considering possible exposures;
(b) comorbidities (risk factors!);
(c) healthcare proxy;
(d) contact data of authorised people, relatives;
(e) physical examination, in particular vital parameters (heart rate/beats per minute, blood pressure, oxygen saturation, respiratory rate)

This interview should identify risk factors for a severe course of disease:

(a) older age (≥ 50 years; mortality is greater than 15% if age >80 years);
(b) smoking;
(c) pre-existing illnesses (cardiovascular system, for example coronary heart disease and primary hypertension; pulmonary disorders, for example asthma, chronic bronchitis; chronic hepatitis; diabetes mellitus; cancer; immune suppression).

#### Diagnostic measures on the Psychiatric COVID Ward

See Appendix 3 for the diagnostic measures used on the ward.

#### Treatment strategies on the Psychiatric COVID ward

The treatment regime is dictated by the illness severity and consists mainly of supportive measures in close consultation with internal medicine specialists. In case of aggravated, severe or critical courses, patients need to be transferred to an internal medicine or intensive care unit. The treatment of the psychiatric disorder should be maintained or, if that is not possible, resumed as soon as possible after discharge from the internal medicine or intensive care unit. The criteria for in-patient treatment or admission to a COVID ward are as follows:

(a) temperature >38 °C;
(b) breathing frequency >20/min, reduced oxygen saturation at room temperature;
(c) systolic blood pressure <90 mmHg;
(d) disorientation;
(e) comorbidities.

See Appendix 4 for details of the treatment strategies on the ward.

#### Additional therapy concepts

A COVID-19 psychosocial support concept was developed at the University Hospital by an interdisciplinary team of psychiatric, psychological, spiritual care, psycho-oncological and palliative care specialists. The new psychosocial support model has been implemented for in-patients with COVID-19, family members and hospital staff members and consists of five elements.^3^ The concept integrates innovative and sustainable ideas, for example, telemedicine-based approaches, and highlights the importance of multidisciplinary collaboration to cope with challenges in the healthcare system.

#### Course of the disease

When treating patients with COVID-19 in a psychiatric unit, severe and critical cases must be detected at an early stage. Early warning indicators for severe disease progression are already known and should be closely observed to avoid complications.

##### Predictors of a severe course of disease

(a) Progressive reduction of lymphocyte count in peripheral blood.
(b) Steady increase of proinflammatory cytokines, for example interleukin (IL)-6 and C-reactive protein.
(c) Steady increase of lactate values.
(d) Steady increase of pathological changes in the lungs within a very short period of time.
(e) Worsening of hypoxaemia under standard oxygen therapy.
(f) Sepsis: temperature >38 °C or <36 °C; heart rate >90/min, breathing frequency >20/min or partial pressure of carbon dioxide <32 mmHg, leucocytes >12 000/mm^3^ or <4000/mm^3^ or >10% immature forms.

##### Criteria for transfer to an internal medicine ward

(a) Transfer patients with the following monitoring indications: cardiovascular monitoring, invasive blood pressure monitoring and/or high-flow oxygen therapy.
(b) Severe clinical course, oxygen saturation <90%, tachypnoea, breathing frequency >24/min, partial pressure of oxygen <70 mmHg, newly emerging arrhythmias, newly emerging pericardial effusion, newly emerging cardiac insufficiency, pulmonary oedema, congested liver or peripheral oedema.

##### Criteria for transfer to an intensive care unit

Rapid respiratory deterioration is an indication for ventilation, catecholamine support and heavy sedation.

#### Further procedures

As soon as patients are sufficiently stable and no longer infectious, they can be discharged from the in-patient setting. However, doctors are often uncertain about the criteria for discharge. The following clinical stability criteria for discharge were established at the LMU University Hospital.

(a) Afebrile ≥3 days.
(b) Symptom onset ≥7 days.
(c) Clinical improvement of respiratory symptoms.
(d) Continuous improvement of COVID-19 lab parameters.
(e) Two negative respiratory SARS-CoV-2 PCR tests at an interval of at least 24 h.

## Discussion

The MPCPCP has enabled the Department of Psychiatry and Psychotherapy at the LMU University Hospital to maintain medical healthcare for patients with mental disorders. It has offered the necessary flexibility to adapt its implementation to the first and second waves of the COVID-19 pandemic in Germany.

Psychiatric patients are considered to be extremely vulnerable in a pandemic for a multitude of disease-specific, comorbidity-related and sociodemographic reasons.^1^ Additional stressors, such as curfews, quarantine and isolation, and the stress of the overall situation during a pandemic can lead to a distinct increase in psychological stress, not only in people with mental disorders but also in the entire general population. On the basis of the experience of the Department of Psychiatry and Psychotherapy at the University Hospital of LMU Munich, we argue that psychiatric hospitals/units can not only contribute to the care of patients with mental disorders with SARS-CoV-2 infection but must also attend to non-psychiatric COVID-19-positive patients who need admission to hospital but not intensive care. The MPCPCP offers guidance on how to position psychiatry in such critical and challenging times. It also illustrates how psychiatric units or hospitals can uphold their mission to care for and protect highly vulnerable population groups, i.e. people with mental illness, by keeping state-of-the-art psychiatric services up and running instead of closing them and at the same time offering relief to internal or emergency medicine departments that are flooded with patients. In summary, psychiatric services are an essential part of medicine, a fact that does not change during a pandemic; on the contrary, we believe that they are as important or even more important during the onslaught of a pandemic like COVID-19.

### Limitations

One limitation of the pandemic plan presented here is that it applies primarily to university hospitals or hospitals where cooperation between psychiatry and internal medicine or tertiary care units is assured. These relationships are important to allow patients with severe COVID-19 disease progression to be transferred at an early stage to an internal medicine or intensive care unit. This point leads to the second limitation, i.e. the applicability of MPCPCP is limited, especially regarding the establishment of a COVID ward; other measures can be completely adapted to other settings.

## Data Availability

Data availability is not applicable to this article as no new data were created or analysed in this study.

## Supplementary material

For supplementary material accompanying this paper visit https://doi.org/10.1192/bjo.2020.167. click here to view supplementary material

## Appendix 1 Synopsis of symptoms, course of disease and diagnostic criteria of severe acute respiratory syndrome coronavirus 2 (SARS-CoV-2) infection

*Clinical symptoms*

(a) Fever (88%).
(b) Unproductive/dry cough (68%).
(c) Indisposition/exhaustion (38%).
(d) Dyspnoea (19%).
(e) Gastrointestinal symptoms (nausea, vomiting, diarrhoea) (5%).
(f) Change of taste or smell (documented by the Robert Koch Institute).

Further symptoms: headache and limb pain, anorexia, weight loss, swelling of lymph nodes, apathy, fatigue, somnolence, confusion. *Stages of disease*

(a) Replicative phase: viral replication in throat area. Duration: several days. Relatively mild symptoms as a result of direct viral cytopathic effect and innate immune response.
(b) Adaptive immune response: frequently, reduces virus load; less frequently, immune response is aberrant, with tissue damage and rapid clinical worsening.

*Course of disease*

(a) Incubation period: 5–6 days (range 1–14 days).
(b) 80% mild to moderate: patients with or without pneumonia, without dyspnoea, with oxygen saturation ≥90%, pulmonary infiltrates in less than half the lung.
(c) 14% severe with dyspnoea, breathing frequency >30/min, oxygen saturation <90%, pulmonary infiltration in more than half of the lung, yet not life-threatening.
(d) 6% critical to life-threatening with respiratory failure, septic shock, multiple organ failure.^4^

*Diagnosis*

(a) *Either* specific: viral pneumonia; *or* unspecific: acute respiratory symptom of any severity.
(b) Lab diagnosis: direct pathogen detection by sample isolation (culture) or nucleic acid detection.
(c) Epidemiological confirmation: epidemiological link with proven infection.
(d) Occurrence of two or more pneumonias in one medical institution with probable or suspected epidemiological link, even without direct pathogen detection.

Diagnostic criteria Diagnosis coronavirus disease 2019 (COVID-19): positive polymerase chain reaction test for SARS-CoV-2 (regardless of material) Suspected COVID-19:

(a) typical clinical symptoms;
(b) typical computed tomography changes;
(c) eosinopenia.

## Appendix 2 Course of h1action in patients with and without mental disorders and severe acute respiratory syndrome coronavirus 2 (SARS-CoV-2) infection: Munich Psychiatric COVID-19 Pandemic Contingency Plan – detailed approach and procedures

**Table:**

	General measures	Out-patient care	In-patient care	Therapies	Staff	COVID ward
Phase I	Events postponed Teaching cancelled Electroencephalogram (EEG)/ electrocardiogram (ECG) ✓ ECT ✓	Hygienic measures Separate room, wearing of face mask Screening questions about possible SARS-CoV-2 infection at first contact	Hygienic measures No therapy restrictions	Psychotherapy/occupational therapy/ physical therapy/ social services/ addiction treatment/ supervision: fully available	Hygienic measures Staff with risk region contacts: 14-day quarantine	−/−
Phase II	Patient contact restricted Visiting ban EEG/EKG ✓ ECT ✓	Emergencies Cases that prevent in-patient admissions	Reduction of therapy group size (2 m distance!) Reduction of therapy content (no sports, cooking and so on)	Psychotherapy: groups across wards cancelled, groups within wards reduced (2 m distance!), individual sessions where possible Occupational therapy/physical therapy: 1 session/week Social services: available, 2 m distance, hygienic measures, no bodily contact Addiction therapy limited to detoxification Supervision	Protective mask obligatory Back-up rules for doctors Home office options	−/−
Phase III	Protective mask obligatory ECT ✓ Polymerase chain reaction (PCR) testing for staff and patients	Entry controls, screening	Isolation measures in suspicious cases Preparation of Psychiatric COVID ward	Reduced multimodal therapy programme, see phase II Telemedicine psychotherapy Crisis management via telephone	Protective mask obligatory Team-building preparations/ emergency Team A: direct patient care; Team B: home office Mobilisation of medical students Crisis intervention for staff via telephone Daily conference calls for staff	Close interaction with internal ward Daily testing available Oxygen administration available
Phase IV	Consideration of ethical issues Patients provision, healthcare proxy Dealing with fatality/death	See phase III		Further reduction of multimodal therapy programme Telemedicine for basic psychotherapy	Crisis intervention for staff via telephone	COVID ward: Mental disorder plus positive SARS-CoV-2 diagnosis PCR testing
				Multimodal therapy programme cancelled Preparation for psychosocial rehabilitation ward	Shifting of staff	Mass epidemic: Any disorder plus positive SARS-CoV-2 diagnosis Internal medicine care prioritised
				−/−	Further shifting of staff	Emergency plan: coping with high numbers of patients
Phase V	Phase V is considered as a long-term period (>2 years). Patients with SARS-CoV-2 infection are expected to continuously present at the Psychiatric Hospital					

## Appendix 3 Diagnostic tests on the Psychiatric COVID ward

**Table:**

Laboratory examination	*Lab tests at admission*: differential blood count, C-reactive protein (CRP), procalcitonin (PCT), interleukin (IL)-6, kidney and liver values, troponin T, pro-brain natriuretic peptide, immunoglobulin, lymphocyte subpopulation, lactate dehydrogenase (LDH), ferritin, coagulation, D-dimer, lactate, beta-human chorionic gonadotropin in female patients of childbearing age. *Daily lab tests*: depend on clinical symptoms, important for course of disease: PCT, IL-6, D-dimer, ferritin, differential blood count, liver values, troponin T.
	*Typical changes* (a) Leucopenia, lymphopenia: frequently, thrombocytopenia (mild) in up to 35% of patients, eosinopenia in up to 52%. (b) CRP increase (in 61–86%) (>10; mostly 10 to 20). (c) LDH increase (in 27–75%). (d) PCT increase (in 24%, especially in patients in intensive care units). (e) Aspartate aminotransaminase/alanine aminotransaminase increase (in 4–22%). (f) Troponin increase.^5^
	Caveat: Cardiomyopathy with troponin increase is a predictor for increased mortality, especially if troponin increases continuously from day 4. Troponin control is strongly recommended. Troponin increases seem to be connected to a COVID-19-associated cardiomyopathy rather than a myocardial infarction.
Medical imaging	(a) In conventional chest X-ray, changes are visible in 50–60% of patients. However, in computed tomography (CT) scans changes are visible in approximately 85% of cases as ground glass opacity (GGO), bilateral, less frequently unilateral infiltration (14–25%) or increase of interstitial markings (Source: Robert Koch Institute, https://www.rki.de). (b) Chest CT (non-contrast): at admission and for further assessment of course of disease in case of clinical worsening. Long-range indication advisable! (c) CT has higher sensitivity than chest X-ray: chest X-ray abnormal in >60% of patients, CT abnormal in >85%. (d) Typical changes: GGO; bilateral, less frequently unilateral infiltration (14–25%), possible increase of interstitial markings.
Virological examinations	(a) Polymerase chain reaction (PCR): most sensitive non-invasive test. (b) First sample: PCR from deep nasal/throat swab; sensitive in first week (replicative phase in throat area). In case of negative test result but continued clinical suspicion, a second test is advisable. (c) Second sample: lower respiratory passages, for example tracheobronchial secretion (extraction, no bronchoalveolar lavage) or sputum. If pulmonic-infiltrated sputum is detected, then nasal/throat swab (progressive disease). In case of typical clinical picture (and CT) and negative PCR, isolation should be maintained and test repeated. (d) Optional: Influenza A/B and respiratory syncytial virus (quick test). (e) In case of clinical suspicion: sputum bacteriology + mycoplasma + sputum for extensive PCR. (f) Legionella urinary antigen test. (g) Organ- or bone marrow-transplanted/immune-suppressed patients (tumour necrosis factor inhibition): Epstein–Barr virus/cytomegalovirus PCR, adeno-PCR. (h) Aspergillus antigen and beta-glucan in case of clinical suspicion. (i) Blood cultures in case of fever and before empirical antibiosis.

## Appendix 4 Treatment strategies on the Psychiatric COVID ward

**Table:**

General treatment measures	Top priority: hygiene measures and immediate isolation! Symptomatic treatment (a) Moderate volume loading for circulatory stabilisation: restrictive volume management – seek negative fluid balance: consider worsening of oxygenation. (b) Antipyretic medication: paracetamol, ibuprofen. (c) Respiratory therapy: respiratory exercise, expectorating, sodium chloride inhalation.
Supportive therapy	(a) Deep vein thrombosis prophylaxis (enoxaparin sodium 40 mg), if no oral anticoagulation. (b) Intermittent proton pump inhibitor if not urgently indicated (Ulcer? Gastrointestinal bleeding? Gastro-oesophageal reflux disease?). (c) Continue antihypertensives (angiotensin converting enzyme (ACE) inhibitors, do not pause or start administration of angiotensin II type 1 blocker). (d) Breathing exercises, physical mobilisation by nursing staff if possible, Thera-Band®, physiotherapy. (e) Nutrition, especially in patients with disease-related anorexia.
Administration of oxygen	(a) Oxygen administration: preferably cautiously (infectiousness may increase through aerosol formation during oxygen administration); only in distinct dyspnoea in clinically symptomatic patients. (b) Aim: oxygen saturation of 90–95% at breathing frequency <20/min. (c) Oxygen administration if oxygen saturation <92% (no later than 90%). (d) Nasal, mask or high-flow oxygen, as needed (Caveat: increased aerosol formation). Caveat: silent hypoxaemia: persisting breathing frequency >20/min; oxygen demand ≥4 L/min, seek transfer to intensive care unit; in patients with sudden deterioration or persistent breathing frequency >30/min, early fasting is advisable.
Special issues	*Antibiotics*: only in case of suspected bacterial superinfection – signs: clinical worsening, sudden increase in C-reactive protein, leucocytosis. Procedure (a) Take several blood samples (aerobic + anaerobic). (b) Consider sputum culture. (c) Check lab parameters (C-reactive protein, procalcitonin test, differential blood count). Calculated antibiotic therapy As per guideline: community-acquired pneumonia Amoxicillin/clavulanic acid 3 × 875/125 mg (taken orally) or ampicillin/sulbactam 3 × 3 g i.v. ± azithromycin 1 × 500 mg i.v. Consider combination of azithromycin or moxifloxacin (Caveat: interactions and QTc interval). Azithromycin and other macrolide antibiotics (additional immune modulatory effects) can be preferably administered in patients with coronavirus disease 2019 (COVID-19) with pulmonary involvement.
	Caveat: prophylactic administration of antibiotics without proof of bacterial infection is not recommended. In patients with suspected bacterial superinfection and/or septic course, calculated antibiotic therapy should be initiated, in case of sepsis within 30 min. In case no pathogens are detected and procalcitonin level is in reference range, antibiotic therapy should be terminated within 48 h.
	*Corticosteroids*: no corticosteroid administration without distinct indication.
	*Antiviral therapy* (a) Currently no antiviral therapy with confirmed efficacy is available.^6^ (b) Uncomplicated patients (in-patient or out-patient): no antiviral therapy. (c) Preferred treatment in clinical studies and under careful consideration of risk–benefit balance and in intensive care units.
	*ACE inhibitors*: continue ACE inhibitors and angiotensin receptor blockers but do not start new administration
Management of psychotropic medication	*Lithium*: in case of high fever, sufficient intake of fluids, distributed throughout the day. In case of additional vomiting/diarrhoea, dose adjustment; as needed, pause lithium treatment for 24 h. *Clozapine*: (agranulocytosis!) initial continuation of clozapine (treatment success of psychotic disorder not to be jeopardised by discontinuation or dose reduction; check blood count regularly) (taken from the Consensus statement on the use of clozapine during the COVID-19 pandemic).^7^ Caveat: in case of bacterial superinfection, calculated antibiotic treatment (see above), close consultation with the internal medical unit. (a) *Ongoing psychopharmacological treatments* should be prioritised and most doses should be reduced by 25–50% of original dose if the patient receives lopinavir/ritonavir, with some exceptions, including quetiapine, asenapine, olanzapine, sertraline, lamotrigine, bupropion and methadone. (b) If levels of the usual psychopharmacological doses are in the low-to-median range, a dose change is not recommended while COVID-19 drugs are being co-administered. Instead, electrocardiogram (ECG) and clinical monitoring of adverse effects and drug levels, if required. (c) When introducing a psychopharmacological drug, dose titration should be progressive, with ECG monitoring if cardiotoxic interactions are present.^8^
Management of psychiatric comorbidities	(a) In agitated delirium, olanzapine is recommended as the first-line antipsychotic; quetiapine should be avoided. (b) In severe mental illness, essential treatments should be maintained. (c) In non-severe mental illness with depressive/anxiety symptoms, psychological support should be provided and symptoms identified and treated.^8^
