# Supplementary material for: Role of psychiatric hospitals during a pandemic: introducing the Munich Psychiatric COVID-19 Pandemic Contingency Plan
Source: BJPsych Open. 2021 Feb 1;7(2):e41. doi: 10.1192/bjo.2020.167 (PMC7853741; doi:10.1192/bjo.2020.167)
Supplement: Supplementary file 1 [file S2056472420001672sup001.docx]

**Supplementary material**

**Laboratory test methods for employees at the Department of Psychiatry and Psychotherapy**

To detect the presence of SARS-CoV-2 in throat-swab specimens, we performed commercial real-time polymerase chain reaction (PCR) assays with the TaqMan 2019-nCoV Assay Kit, No. A47532 and the TaqMan 2019-nCoV Control Kit, No. A47533 (Thermofisher Scientific, ‎Waltham, MA, USA). The assay targets three different viral genomic regions and thus reduces the risk of false negatives (ORF1ab, spike [S] gene and nucleocapsid [N] gene). Furthermore, the assays underwent bioinformatic selection and analyses to specifically target sequences that are unique to SARS-CoV-2. Additionally, an RNase P assay was run in duplex with each 2019-nCoV assay as an internal positive control. Finally, the TaqMan 2019-nCoV Assay Kit was used together with the TaqMan 2019-nCoV Control Kit to monitor assay-specific amplification. The nucleic acids used for detecting SARS-CoV-2 were extracted from 50 µL of throat-swab medium with a MagMax 96 Viral RNA Isolation Kit, No. AM1836 (Thermofisher Scientific, ‎Waltham, MA, USA), according to the manufacturer’s instructions. Real-time PCR assays were conducted in a total volume of 20 µL with 5 µL RNA and the TaqMan Fast Virus 1step Master Mix, No. 4444432. Samples were run on the StepOnePlus Real-Time PCR System (Thermofisher Scientific, ‎Waltham, MA, USA). All assays are established procedures in the laboratory of the Department of Psychiatry and were performed according to the manufacturers’ instructions. Supplementary Fig. 1 shows the real-time PCR amplification plot for detecting SARS-CoV-2. The curves around cycle 20 are for the positive controls for ORF1ab, spike (S) gene and nucleocapsid (N) gene. At cycles above 25, positive verification of SARS-CoV-2 can be seen for three tested individuals. The assays for ORF1ab, spike (S) gene and nucleocapsid (N) gene were tested by Thermofisher with related organisms to display the specificity of the design. No amplification was seen for any near neighbour pathogens. In summary, the assays are specific against 2116 other complete genomes of corona viruses in the National Center for Biotechnology Information. In addition, all three assays showed high efficiency (96% to 100%) and sensitivity down to ten copies.


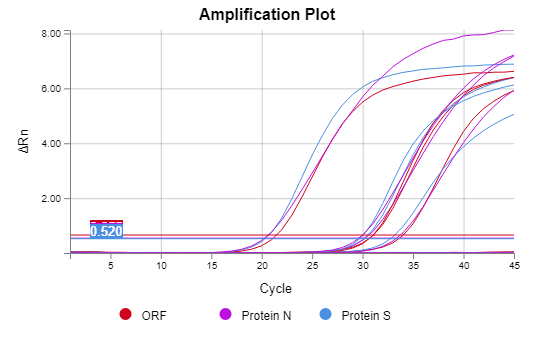


Supplementary Fig. 1 Real-time polymerase chain reaction (PCR) amplification plot for detecting SARS-CoV-2 (x-axis, PCR cycle number; y-axis, signal intensity).
